# Enzymatic Activity Analysis and Catalytic Essential Residues Identification of *Brucella abortus* Malate Dehydrogenase

**DOI:** 10.1155/2014/973751

**Published:** 2014-05-07

**Authors:** Xiangan Han, Yongliang Tong, Mingxing Tian, Yuxi Zhang, Xiaoqing Sun, Shaohui Wang, Xusheng Qiu, Chan Ding, Shengqing Yu

**Affiliations:** Shanghai Veterinary Research Institute, The Chinese Academy of Agricultural Sciences (CAAS), 518 Ziyue Road, Shanghai 200241, China

## Abstract

Malate dehydrogenase (MDH) plays important metabolic roles in bacteria. In this study, the recombinant MDH protein (His-MDH) of *Brucella abortus* was purified and its ability to catalyze the conversion of oxaloacetate (OAA) to L-malate (hereon referred to as MDH activity) was analyzed. Michaelis Constant (K_m_) and Maximum Reaction Velocity (V_max_) of the reaction were determined to be 6.45 × 10^−3^ M and 0.87 mM L^−1 ^min^−1^, respectively. *In vitro* studies showed that His-MDH exhibited maximal MDH activity in pH 6.0 reaction buffer at 40°C. The enzymatic activity was 100%, 60%, and 40% inhibited by Cu^2+^, Zn^2+^, and Pb^2+^, respectively. In addition, six amino acids in the MDH were mutated to investigate their roles in the enzymatic activity. The results showed that the substitutions of amino acids Arg 89, Asp 149, Arg 152, His 176, or Thr 231 almost abolished the activity of His-MDH. The present study will help to understand MDH's roles in *B. abortus* metabolism.

## 1. Introduction

*Brucella abortus* (*B*.* abortus*), the causative agent of bovine brucellosis, is the most widespread pathogen which results in abortion in pregnant cattle and undulant fever in humans [1–3].* Brucellae* are intracellular pathogens which can survive within a variety of cells including macrophages and maintain a long-lasting interaction with the host cells. Virulence of* Brucella* depends on survival and replication properties in host cells [4].

**Figure 1:**
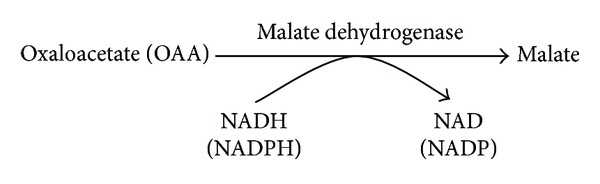
Schematic reaction cycle of catalyzing the conversion of oxaloacetate (OAA) to L-malate by MDH.

Malate dehydrogenase (MDH), a key enzyme in the tricarboxylic acid cycle (TCA), plays important metabolic roles in aerobic energy producing pathways and in malate shuttle (Figure 1) [5]. The enzyme is widely distributed in animals, higher plants, and microorganisms. Until now, MDHs have been isolated and characterized from numerous eukaryotic species, including fungi [6], plants [7], and animals [8]. The three-dimensional structures reveal that MDHs are homomeric enzymes organized usually as either dimers or tetramers with subunit molecular masses of 30–38 kDa, which have homologous active sites, cofactor-binding sites, and quaternary structures based on the sequence comparisons and bioinformatics analysis. However, the kinetic and biochemical properties of bacterial MDHs are less well studied, compared to the enzymes from animals and plants [9]. Recent studies reported that* B*.* abortus* MDH was an important antigenic protein during the infection [10] and vaccination with bacterial-expressed* B*.* abortus* MDH enhanced the protection to* Brucella* infection [11]. However, there has been no report regarding enzymatic and biological properties of* B. abortus* MDH. In this study, we cloned and expressed* B. abortus* MDH and then studied the enzymatic activity and catalytically essential residues of MDH, which will help to understand its roles in* B. abortus* metabolism.

## 2. Materials and Methods

### 2.1. Bacterial Strains, Plasmids, and Growth Conditions

*B. abortus* A19 was obtained from Chinese Veterinary Culture Collection Center (CVCC) and cultured in Tryptic Soy Agar (TSA, Difco, NJ) or Tryptic Soy Broth (TSB, Difco) at 37°C with 5% CO_2_.* Escherichia coli* strains DH5*α* (Invitrogen, Carlsbad, CA) and BL21 (DE3) (Stratagene, La Jolla, CA) were cultured at 37°C in Luria Bertani (LB) medium containing kanamycin at 50 *μ*g/mL or ampicillin at 100 *μ*g/mL as needed. The expression vector pET-28a was purchased from Novagen (Madison, WI). Restriction enzymes were from MBI Fermentas (Hanover, MD). Human plasminogen, mouse monoclonal anti-plasminogen, human fibronectin, mouse monoclonal anti-fibronectin, and HRP-labeled anti-mouse IgG were from Sigma (St. Louis, MO). All chemicals used in this study were of analytical grade and purchased from Sigma.

### 2.2. Cloning and Expression of* B. abortus* MDH

Chromosomal DNA of* B. abortus *A19 was isolated as previously described [12]. The encoding gene,* malate dehydrogenase* (*mdh*), was amplified by polymerase chain reaction (PCR) using primers MDH-F (5′ CGCGGATCCATGGCACGCAACAAGATTG 3′) and MDH-R (5′ CGCGTCGACTTATTTCAGCGACGGAGCA 3′) and subjected to the sequence analysis. The primers were designed according to* B. abortus *S2308* mdh *gene sequence (AM040264.1) with* Bam*HI and* Sal*I site (underlined) inserted. PCR product was* Bam*HI/*Sal*I-digested and cloned into* Bam*HI/*Sal*I sites of pET-28a (+) (Novagen). The resulting plasmids, pET-28a-MDH, was used to transform* E. coli* BL21. Expression of His-tagged MDH (His-MDH) was induced by 1 mM IPTG, analyzed by sodium dodecyl sulfate polyacrylamide gel electrophoresis (SDS-PAGE) and Coomassie blue staining. The recombinant protein His-MDH was purified using HisTrap chelating high-performance columns (Amersham Pharmacia Biotech, Piscataway, NJ) and was quantitatively determined using a BCA protein assay kit (Pierce, Rockford, IL).

### 2.3. Enzymatic Activity and Kinetics Assay of the His-MDH

His-MDH activity was determined by measuring the conversion of OAA to L-malate at 40°C as previously described with modifications [6]. A standard assay consists of adding 1.5 mM OAA and 0.32 mM NADH or NADPH to 2.0 mL phosphate buffered saline (PBS, pH 7.4); 1 *μ*g of His-MDH was then added to initiate the reaction. The reaction was monitored spectrophotometrically by measuring absorbance at 340 nm for the reduction of OAA at 1 min intervals for 5 min at 40°C.

To study the enzymatic kinetics of His-MDH, varying concentrations of OAA substrate (0.19, 0.38, 0.75, 1.50, or 3.00 mM) were used for the assay. The reaction was monitored spectrophotometrically by measuring absorbance at 340 nm for the reduction of OAA at 1 min intervals for 5 min at 40°C. Michaelis-Menten kinetics showed that His-MDH was able to fully convert OAA to L-malate at all five substrate concentration levels. *V*_max⁡_ and *K*_m_ for His-MDH were determined from double-reciprocal Lineweaver-Burk plots.

### 2.4. Effects of pH, Metal Ions, and Temperature on MDH Activity

Modifications to the standard spectrophotometry enzyme kinetics assay were made to study the effects of pH, temperature, and metal ions on His-MDH activity. As for the pH experiment, reaction buffers with pH values ranging from 3.5 to 9.0 in 0.5 increments were used for the assay. For the temperature experiment, reaction buffers prewarmed between 20°C and 70°C at 5°C increments prior to the assay were used for the assay. To investigate the effect of metal ions on MDH activity, 0.5 mM of NiSO_4_, MnSO_4_, MgSO_4_, SnSO_4_, CrCl_3_, PbCl_2_, ZnSO_4_, or CuSO4 was added to the enzyme solution and incubated for 1 h at 40°C, respectively. All the reactions were performed in triplicate.

### 2.5. Sequence Comparisons and Bioinformatics Analysis

Alignment of the amino acid sequences of* malate dehydrogenase* from* E. coli* K-12 (access number P61889),* Salmonella enterica *(*S. enterica,* access number YP_002245258),* Thermotoga maritime* (*T. maritime,* access number P16115),* Archaeoglobus fulgidus* (*A. fulgidus,* access number Z85985), and* B. abortus* (access number NC_007618.1) by DNAstar 7.01 version.

Gaps are marked by dashes. Residues important for catalysis, coenzyme binding, and substrate binding are indicated by* asterisks *and numbered according to the site in malate dehydrogenase.

### 2.6. Site-Directed Mutagenesis, Expression, and Enzymatic Activity Analysis of the MDH Mutant Forms

In order to determine the critical amino acids to the catalytic activity of MDH, several single amino acid substitutions were introduced into the plasmid pET-28a by a modified PCR-based site-directed mutagenesis method [13]. Target primers for generating R89L, D149V, R152L, H176P, D178V, and A231V mutations are given in Table 1. In the primers designed above, codon R89 (CGC) and R152 (CGC) were replaced by CTC (L), codon D149 (GAC) was changed to GTC (V), and codon H176 (CAC) was replaced by CCC (P); the codon D178 (GAU) and A231 (GCU) were substituted by GTT (V). The mutations of the* mdh* gene were confirmed by DNA sequencing and then expressed in* E. coli* BL21 (DE3) as described above. The resulting His-MDH mutant forms were analyzed for the MDH activity by measuring the conversion of OAA to L-malate at 40°C as described above.

### 2.7. Statistical Analysis

Inhibition frequencies of adherence and invasion were expressed as the mean ± standard deviation of *n* independent values. The statistical significance of the differences between mean values was determined using the Student's *t*-test (*P* < 0.05).

## 3. Results

### 3.1. Expression and Purification of* B. abortus* A19 MDH

**Figure 2:**
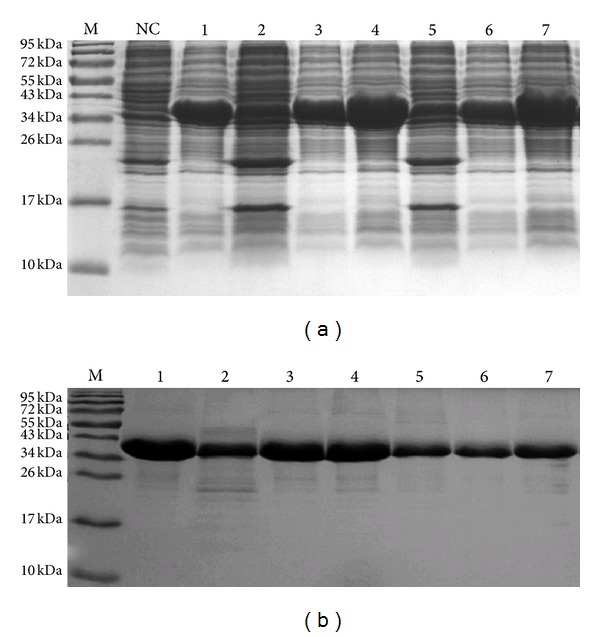
Expression and purification of* B. abortus* MDH and the six mutant forms were identified with SDS-PAGE followed by Coomassie blue staining. (a) SDS-PAGE profiles of the expressed recombinant proteins. Lane M: prestained protein marker (SM0671, Fermentas); lane NC: total cellular proteins of* E. coli* BL21 transformed with pET-28a. No band of 38 kDa His-MDH was shown. Lane 1: total cellular proteins of* E. coli* BL21 transformed with expression plasmids pET-28a-MDH. Lanes 2–7: total cellular proteins of* E. coli* BL21 transformed with pET-28a-derived plasmids containing the mutant forms of the* mdh *gene, including Arg 89 to Leu mutant, Asp 149 to Val mutant, Arg 152 to Leu mutant, His 176 to Pro mutant, Asp178 to Val mutant, and Thr 231 to Val mutant, respectively. (b) Coomassie blue staining of the expressed recombinant proteins. Lane M: prestained protein marker (SM0671, Fermentas). Lane 1: purified proteins of MDH. Lanes 2–7: purified proteins of mutant forms MDH, including Arg 89 to Leu mutant, Asp 149 to Val mutant, Arg 152 to Leu mutant, His 176 to Pro mutant, Asp178 to Val mutant, and Thr 231 to Val mutant, respectively.

Recombinant fusion protein His-MDH was successfully expressed in* E. coli *BL21, as shown by a 38 kDa band in the SDS-PAGE analysis followed by Coomassie blue staining (Figure 2(a), lane 1). After purification with HisTrap chelating high-performance columns (Amersham), a single band of His-MDH was identified (Figure 2(b), lane 1).

### 3.2. MDH Activity and Its Influence Factors of pH, Temperature, and Metal Ions

**Figure 3:**
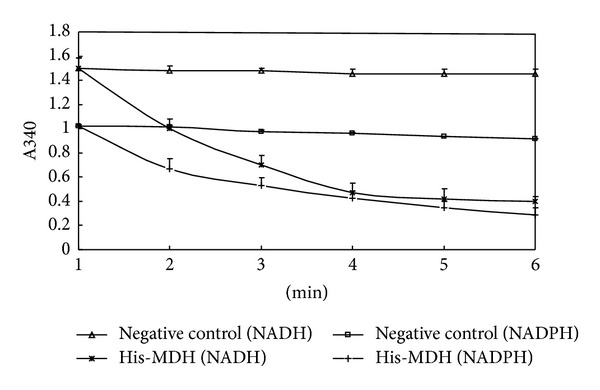
Enzymatic analysis of* B. abortus* His-MDH. One microgram of purified His-MDH was added to the reaction buffer and the enzymatic kinetics was measured. The enzymatic activity was detected as detailed in Materials and Methods. The experiments were performed in triplicate, and the bars represent standard deviations.

His-MDH's activity of catalyzing the conversion of OAA to L-malate was determined by following the change in absorbance at 340 nm. The value of absorbance at 340 nm changed from 1.5 to 0.6 and from 1.0 to 0.3, respectively, during 5 min at 40°C by using NADH or NADPH as cofactor, respectively. The results showed that His-MDH has high catalytic capability under our experimental conditions (Figure 3).

**Figure 4:**
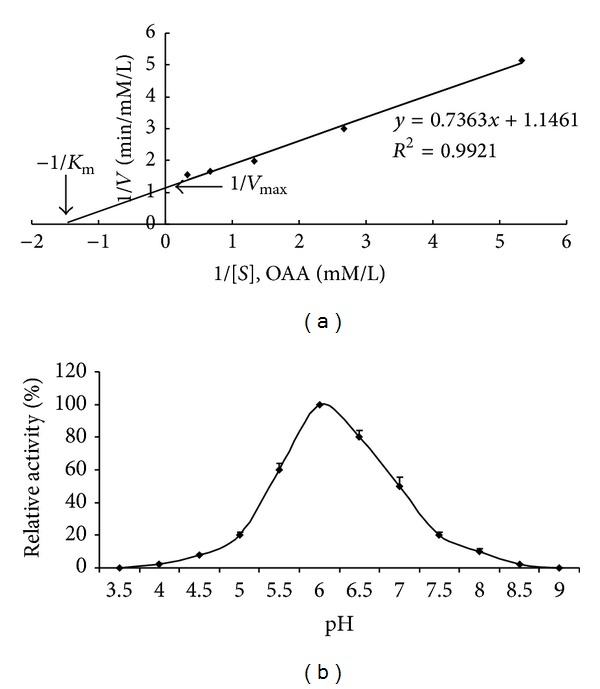
The influence factors of enzymatic activity. (a) The conversion-rate (V) of OAA (*S*) to L-malate was measured at 340 nm using reaction of 1 *μ*g of His-MDH with nd pH and d racterizationvarious concentrations of OAA (0.1875–3 mM) at pH 6.0, 40°C. Measurements were performed at 1 min intervals for 5 min. Data from 3 independent assays were plotted by the method of Michaelis-Menten and fitted to the equation *V* = *V*_max⁡_ · *S*/(*S* + *K*_m_). *K*_m_ and *V*_max⁡_ for His-MDH were determined to be 6.45×10^−3^ M and 0.87 mM L^−1^ min^−1^, respectively, by means of a Lineweaver-Burk plot (double-reciprocal plot). Data shown here were the mean values ± standard deviations. (b) The maximal MDH activity occurred at pH 6.0. The experiments were performed in triplicate, and the bars represent standard deviations.

Michaelis-Menten plot was produced using the His-MDH kinetics data with varying substrate concentrations. The plot was fitted to the equation *V* = *V*_*max*_ *S*/(*S* + *K*_*m*_) (Figure 4(a)); Michaelis Constant (*K*_m_) and Maximum Reaction Velocity (*V*_max⁡_) were determined to be 6.45 × 10^−3^ M and 0.87 mM L^−1^ min^−1^, respectively, indicating high catalytic capability of His-MDH for the dehydratation of OAA to L-malate under our experimental conditions.

**Figure 5:**
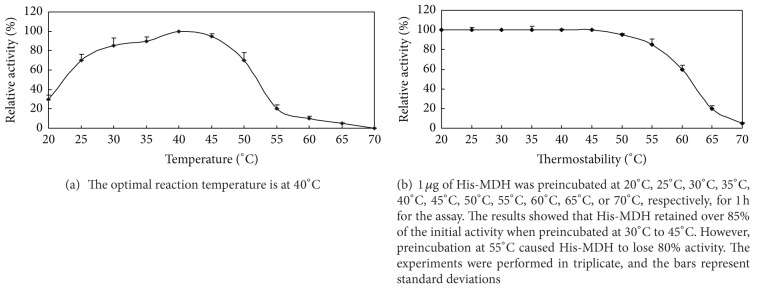
The thermostability of His-MDH.

The optimal pH of His-MDH for OAA reduction was observed to be pH 6.0 (Figure 4(b)). Specific activity was measured at temperature from 20°C to 70°C and was highest at 40°C (Figure 5(a)). His-MDH retained over 85% of the initial activity when preincubated at 30°C to 45°C. However, preincubation at 65°C caused His-MDH to lose 80% activity (Figure 5(b)). Comparing to PBS buffer, His-MDH retained over 90% of the enzymatic activity when adding Ni^2+^, Mn^2+^, Mg^2+^, Sn^2+^, or Cr^3+^ into PBS buffer. The relative activity of His-MDH was reduced to 60% or 40% when Zn^2+^ or Pb^2+^ was added into the PBS buffer, respectively. When Cu^2+^ was added, the enzymatic activity was completely inhibited (Table 2).

### 3.3. Sequence Comparisons and Bioinformatics Analysis

**Figure 6:**
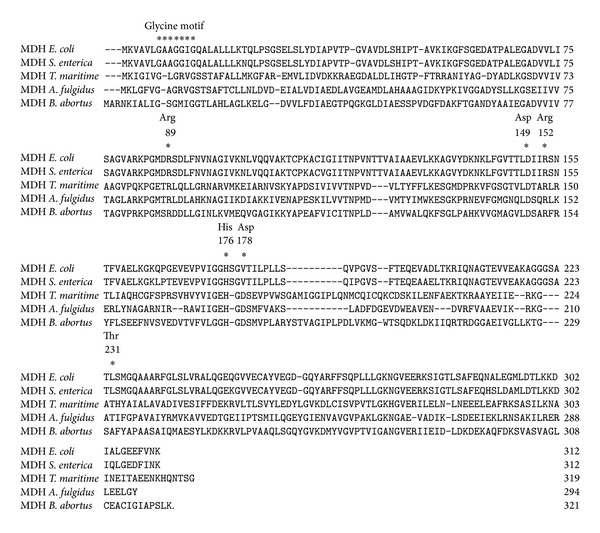
Sequence comparisons and bioinformatics analysis. Alignment of the amino acid sequences of MDH from* E. coli* K-12 (access number P61889),* S. enterica *(access number YP_002245258),* T. maritime* (access number P16115),* A. fulgidus* (access number Z85985), and* B. abortus* (access number NC_007618.1) by DNAstar 7.01 version. Gaps are marked by* dots*. Residues important for catalysis, coenzyme binding, and substrate binding are indicated by* asterisks *and numbered according to the system used for lactate dehydrogenase.

Sequence homology analysis reveals that the deduced amino acid sequence of MDH (321 amino acids) from* B. abortus* shares conserved motifs and acidic residue identity with MDHs from other bacteria, including glycine motif, Arg 89, Asp 149, Arg 152, His 176, Asp178, and Thr 231 (Figure 6).

### 3.4. Expression and MDH Activity Analysis of the His-MDH Mutant Forms

Six single amino acid substitutions were introduced into the plasmid pET-28a by modified PCR-based site-directed mutagenesis. The recombinant His-MDH mutant forms were successfully expressed in* E. coli *BL21 by SDS-PAGE analysis with Coomassie blue staining (Figure 2).

**Figure 7:**
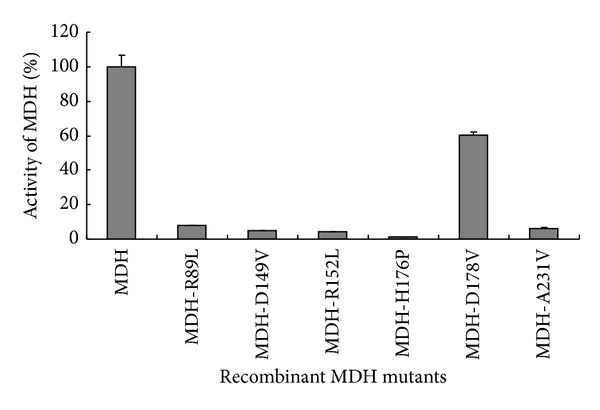
Enzymatic activity of the MDH mutant forms. The activities of the six mutant forms of the MDH were detected by measuring absorbance at 340 nm for the reduction of OAA at 1 min intervals for 5 min at 40°C. The results showed that the substitutions of amino acids Arg 89, Asp 149, Arg 152, His 176, and Thr 231 almost abolished the activity of MDH. However, the mutant form with the substitutions of amino acids Asp178 kept 60% activities of the original MDH. The experiments were performed in triplicate.

The activities of these mutant proteins were measured according to the assay described above. The results showed that the substitutions of amino acids Arg 89, Asp 149, Arg 152, His 176, and Thr 231 almost abolished the activity of MDH. However, the activity of the mutant form with the substitutions of amino acids Asp178 kept 60% activities of the His-MDH (Figure 7).

## 4. Discussion

ngativeIn this study,* B*.* abortus* MDH was cloned and expressed successfully. The His-MDH can catalyze the conversion of OAA to L-malate by using NADH or NAPDH as cofactor, which will help in* B*.* abortus* survival* in vivo*. The optimum activity of the expressed His-MDH was found with temperature at 40°C and PH 6.0. The optimum temperature was in accord with that of* F. frigidimaris *MDH (40°C) but was much lower than the responding values of bacterial MDHs from* E. coli*,* Bacillus stearothermophilus*, and* Salinibacter rubber* (50–67°C) [14]. MDH activity of* B*.* abortus* had a relatively broad range from 30°C to 45°C, which retained over 85% of the activity. The result of optimum pH indicated that the acidic condition (pH = 6) is more suitable to MDH activity. The optimum high reaction temperature and low pH for* B. abortus* MDH activity might be due to the structure and characteristics of MDH and beneficial in prompting the process of infection [15]. The MDH activity was strongly inhibited by Cu^2+^, Zn^2+^, and Pb^2+^, resulting in 100%, 60%, and 40% reduction of the maximal activity, respectively. The results showed that the enzymatic activity of His-MDH is ion dependent. The results were in agreement with that previously published data on MDH activities of* Streptomyces avermitilis* [14]. MDH is reported as an important enzyme of TCA, which plays important metabolic roles in aerobic energy producing pathways. Furthermore, our previous results showed that MDH is related to* B. abortus* pathogenesis and may act as a new virulent factor [16]. Our results imply that inhibiting MDH activity using Cu^2+^, Zn^2+^, or Pb^2+^ to block the TCA metabolism pathway might become a new way to control* B. abortus* infections.

In this study, the MDH amino acid sequence from* E. coli* K-12,* S. enterica*,* T. maritime*,* A. fulgidus*,and* B. abortus* was selected and aligned. Based on the sequence comparisons and bioinformatics analysis, highly conserved residues within MDH family, including catalysis, coenzyme binding, and substrate binding, are selected. A glycine motif (GXGX_2_GG) is identified in the N-terminal sequence of MDH from* B. abortus*. This motif is highly conserved within MDH family and involved in the binding of NAD^+^ [17]. The crystal structure of MDH from* B*.* abortus* is analyzed in NCBI (http://www.ncbi.nlm.nih.gov/Structure/mmdb/mmdbsrv.cgi?uid=70885); the results showed that MDHs are homomeric enzymes organized usually as tetramers with subunit molecular masses of 34 kD, the resolution is 2.3 Å, and each MDH subunit bound NAD^+^ as cofactor, which is in accord with the previous results that all MDHs are NAD^+^ dependent except the chloroplast enzyme, which requires NADP^+^ as a cofactor [9]. Although some previously studies showed that other MDHs have homologous active sites, cofactor-binding sites, and quaternary structures except glycine motif (GXGX_2_GG), the data concerning MDH of* B. abortus* are very limited. Consequently, in this study, for further study the active sites of* B. abortus* MDH, six putative critical amino acids (including Arg 89, Asp 149, Arg 152, His 176, Asp178, and Thr 231), which are involved in MDH catalytic functions, were selected and then substituted with amino acids using site-directed mutagenesis. The results showed that the substitutions of amino acids almost abolished the activity of MDH except Asp178. Further study showed that the amino acids Arg 89, Asp 149, Arg 152, and His 176 of* B. abortus* MDH are necessary for catalysis and conserved among different species MDHs (such as* E. coli* and* A. fulgidus*) [18]; only Thr 231 is specific for* B. abortus* MDH. The results also showed different species MDHs have homologous active sites. Furthermore, those active sites may play a similar catalytic function of MDHs. For example, His 176 and Asp-149 form a proton relay system, while Arg-89 stabilizes the polarized carbonyl bond of the substrate during the transition state. Arg-152 binds and orients the substrate [9, 19].

The previous studies showed that* B*.* abortus* MDH was an important antigenic protein, which plays important roles during the infection [10]. Furthermore, vaccination with bacterial-expressed* B*.* abortus* MDH enhanced the protection to* Brucella* infection. Concerning the importance of metabolic roles of MDH in aerobic energy pathways and malate aspartate shuttle, therefore, newly found MDH active sites from B. abortus show a promising therapeutic target for control of brucelosis [20, 21].

## 5. Conclusions

In conclusion, His-MDH can catalyze from OAA to L-malate. Amino acids Arg 89, Asp 149, Arg 152, His 176, and Thr 231 are critical amino acids to the catalytic activity of MDH. These findings will be beneficial for future research concerning the role of MDH in* B. abortus* pathogenesis and the control strategy of the bacteria.

## Figures and Tables

**Table 1:** Primers used for site mutation.

Plasmid	Primer^a^	Sequence (5′~3′)^b^	Purpose	Mutagenesis amino acid (codon)
pMDH	MDH-F	CGCGGATCC ^c^ ATGGCACGCAACAAGATTG	Gene cloning	
	MDH-R	CGCGTCGAC ^d^ TTATTTCAGCGACGGAGCA	Gene cloning	
pMDH-R89L	MDH-R89LF	GGCATGAGC**CTC**GACGATCTCCTGGGC	Site 89 Arg mutation	Arg(CGC) → Leu(CUC)
	MDH-R89LR	GCCCAGGAGATCGTC**GAG**GCTCATGCC	Site 89 Arg mutation	Arg(CGC) → Leu(CUC)
pMDH-D149V	MDH-D149VF	GGC GTT CTC **GTC** AGC GCC CGCTTCCGT	Site 149 Asp mutation	Asp(GAC) → Val(GUC)
	MDH-D149VR	ACG GAA GCG GGCGCT**GAC**GAGAACGCC	Site 149 Asp mutation	Asp(GAC) → Val(GUC)
pMDH-R152L	MDH-R152LF	GACAGCGCC**CTC**TTCCGTTATTTCCTC	Site 152 Arg mutation	Arg(CGC) → Leu(CUC)
	MDH-R152LR	GAGGAAATAACGGAA**GAG**GGCGCTGTC	Site 152 Arg mutation	Arg(CGC) → Leu(CUC)
pMDH-H176P	MDH-H176PF	CTGGGTGGC**CCC**GGCGATTCGATGGTT	Site176 His mutation	His(CAC) → Pro(CCC)
	MDH-H176PR	AACCATCGAATCGCC**GGG**GCCACCCAG	Site 176 His mutation	His(CAC) → Pro(CCC)
pMDH-D178V	MDH-D178VF	GGCCACGGC**GTT**TCGATGGTTCCGCTG	Site 178 Asp mutation	Asp(GAU) → Val(GUU)
	MDH-D178VR	CAGCGGAACCATCGA**AAC**GCCGTGGCC	Site 178 Asp mutation	Asp(GAU) → Val(GUU)
pMDH-A231V	MDH-A231VF	ACCGGCTCG**GTT**TTCTACGCTCCGGCG	Site 231 Ala mutation	Ala(GCU) → Val(GUU)
	MDH-A231VR	CGCCGGAGCGTAGAA**AAC**CGAGCCGGT	Site 231 Ala mutation	Ala(GCU) → Val(GUU)

^a^Amino acids are represented by their one-letter abbreviation, and the number indicates the localization of the mutated residue in the amino acid sequence of MDH.

^b^Mutations that have been introduced in the oligonucleotides of the *mdh* gene are indicated in bold.

^c,d^ *Bam*HI and *Sal*I sites were underlined, respectively.

**Table 2:** Effect of metal ions on MDH activity of *B. abortus*.

Metal ions^a^	Relative activity (%)^b^
None	100
Ni^2+^	98
Mn^2+^	95
Mg^2+^	96
Sn^2+^	95
Cr^3+^	90
Pb^2+^	60
Zn^2+^	40
Cu^2+^	0

^a^Each metal ion was added into the reaction buffer at 0.5 mM of the concentration, respectively. None means no metal ion added.

^b^MDH activity in the reaction buffer without metallic salts was valued as 100%. Relative activity represented the value which compared to that of no additional metal ion added.
